# Device for Acoustic Support of Orientation in the Surroundings for Blind People †

**DOI:** 10.3390/s18124309

**Published:** 2018-12-06

**Authors:** Mariusz Kubanek, Janusz Bobulski

**Affiliations:** Institute of Computer and Information Sciences, Czestochowa University of Technology, 42-201 Czestochowa, Poland; janusz.bobulski@icis.pcz.pl

**Keywords:** signal processing, image processing, bioinformatics, bioengineering, blind people, acoustic space

## Abstract

The constant development of modern technologies allows the creation of new and, above all, mobile devices supporting people with disabilities. All work carried out to improve the lives of people with disabilities is an important element of the field of science. The work presents matters related to the anatomy and physiology of hearing, imaginative abilities of blind people and devices supporting these people. The authors elaborated a prototype of an electronic device that supports the orientation of blind people in the environment by means of sound signals. Sounds are denoted to present to a blind person a simplified map of the depth of space in front of the device user. An innovative element of the work is the use of Kinect sensor, scanning the space in front of the user, as well as a set of developed algorithms for learning and generating acoustic space, taking into account the inclination of the head. The experiments carried out indicate the correct interpretation of the modeled audible signals, and the tests carried out on persons with impaired vision organs demonstrate high efficiency of the developed concept.

## 1. Introduction

The Device for Acoustic Surround Orientation for Blind People (DASOBliP) [1] is a prototype device designed to support blind people navigate inside and outside buildings by hearing sound signals, which enclose information about distances to obstacles in diverse points of the distance map take over by the sensor—Xbox Kinect.

A blind person will be able to orientate in environment by receiving a series of audio signals that give her information about the place and the distance to which the obstacle is located (or not). Taught to properly interpret the signals of the blind person’s brain, based on the information provided ought to start to reflexively process sound information, thanks to which the blind person will start to receive the surroundings as if he saw them.

The distance map of the area in front of a blind person, captured by the Xbox Kinect, is processed by a mini Raspberry Pi computer, which plays relevant sound signals (previously generated and saved to files) at the appropriate volume for right and left channels. The reproduced sounds are played by means of the earphones. The sensor and computer power supply was realized using a power bank. The operating system, program and sound files used by the computer are stored on memory card at the tenth speed class. The sensor has been fastened to a bicycle helmet (having only the function of stable fit to the head) using a special TV holder for the Kinect sensor.

## 2. Spatial Hearing

Man can locate a sound source with great precision. Hearing is possible because of several phenomena, such as the difference in the intensity of sound between ears, the inter-temporal differences of time, and also thanks to spatial filtering [2].

The differences in the volume of sound between ears are not so important in locating the sound source, if they are lower than 2000 Hz (single tone) or 3000 Hz (chord), because below these frequencies they are too small. This is due to the weak suppression of low tones through the head. However, when the sound has a higher frequency, then the difference in the intensity of sound in both ears begins to be useful. Such sound frequencies are already effectively suppressed by the head. The location of the source of signal based on the difference in intensity is named the duplex theory. This peculiarity works well at high tones, while complex sounds and low tones are located using inter-temporal time differences [2].

Inter-temporal differences of time are the peculiarity of sound phase shift between one ear and the other, caused by the sound reaching one of the ears with a delay in relation to the first one. Thanks to this properties, it is possible to indicate the side from which a tone lower than 1500 Hz reaches or a composite sound (which may contain only high frequencies) a one-time (e.g., crack) or periodically repeated, the frequency of which is less than 600 Hz [2].

The mechanisms described above are not able to determine the exact direction from which the sound arrives. These mechanisms are not able to point whether the sound is coming from above, from behind or from anywhere. Hearing uses one more mechanism, which is termed spatial filtering. Thanks to the earlobes and the head, the spectrum of the sound ear reaches the center. Based on changes in the sound spectrum, it is possible to precisely determine the direction from which the sound comes from. The earlobe only affects sounds higher than 6000 Hz, but the head already has an effect on frequencies from 500 Hz to 16,000 Hz [2].

Each person has an individual characteristic of the above described filtering. Functions characterizing changes in the spectral structure of sounds upcoming from different directions are entitled Head Related Transfer Function (HRTF). The ear cone also helps to determine if the sound comes from inside (e.g., the impression from the headphones) or from the outside [2,3].

### 2.1. Imaginative Ability of Blind People

In the blind, there is no doubt about the presence of spatial imagination. Apart from all scientific research, proof of such imagination are sculptures and convex drawings created by blind people, effective use of touch maps and other situations in which spatial imagination is necessary and in which blind people manage. Furthermore, spatial imagination takes place in people who have never seen, and so have never practiced any visual impressions, but it is not obvious in what form. Some say that there is a visual imagination in such blind people, i.e., the ability to visualize, although others disagree with such a statement and use other terms that they think are more accurate [4].

For the device created to support orientation in the surroundings, an important skill of blind people is the ability to create in the imagination a space model based on a verbal description. Even though the created prototype does not use verbal descriptions of space, the abovementioned capabilities indicate that the blind are sufficiently imaginative to use the system of acoustic support of orientation in the environment, although it will be necessary to use the training to fully use the capabilities of the device.

In addition, there are a number of visualization devices, supported by computer techniques, aimed at helping the blind in contact with the environment, e.g., mapping 3D objects to the bas-reliefs [5,6].

### 2.2. Overview of Hardware and Software Supporting

There are several sorts of devices and software supportive the blind. These include braille printers, Braille watches or with the option of sound hour reading, ordinary white sticks, software overlays for the blind or independent programs—for instance speech synthesizers for text reading or specific versions of GPS software [7]. A particular set of assistive devices blind people are aids orientation in the environment. These are especially intended for the blind GPS device, but also obstacle and objects detectors or imaging devices.

The white stick was probably the first device used by blind people to study the surroundings. The very idea of a cane is much earlier than its white color. It was only later that the white color was used to make the man more visible to other persons. The color itself has also become a symbol informing that the person using the cane is blind [7].

UltraCane device has the form of a white stick, but additionally its handle is armed with ultrasonic detectors of obstacles. The user is informed about the detection of an obstacle (in the direction that he points) through two vibrating elements of the button-shaped handle [8].

‘K’ Sonar is a minor device that the user holds in his hand. The device can be additionally mounted on the handle of a normal white stick, which increases the stability and lets the simultaneous use of the white stick and the device. In this combination, the user should hold the set directly behind the device handle, not sticks. ‘K’ Sonar uses an ultrasonic detector; however, the method of communication with the user and the type of information provided about objects are different than those used in UltraCane [9].

The vOICe is a group of programs that allows anyone interested to submit blind people for imaging device. To make the device you need a smartphone or computer, headphones and a low resolution web camera [10].

BrainPort^®^ V100 is alternative device that “transmitting” the image to a blind person not by means of sounds or vibrations, but using a series of electrodes located on the tongue. Even though device testing has been going on for many years, it was released for use and sale in the United States on 19 June 2015. Currently available in the USA and the EU [11].

The Kinect sensor has also been used in a non-invasive apnea detection system and breath rate observation using real-time image sequence analysis recorded in any sleep position and in all lighting conditions (even in the dark). A Microsoft Kinect sensor was used to visualize changes in the chest and abdomen from the respiratory rhythm [12].

In paper [13], the authors propose an idea similar to ours for helping the visually impaired in recognizing things in the everyday environment. This conception is implemented as a portable device consisting of a Microsoft Kinect sensor, a white stick, a tactile feedback device, and other elements. Using the Kinect sensor, the system searches for an object that the user asks the system to find, and then returns the search result to the user using the tactile feedback device.

In addition to the aforementioned methods using the kinect sensor, there are a number of other systems that use other sensors such as a smartphone [14] and a laser [15] that use other obstacle detection methods.

In the project Espacio Acustico Virtual [16], a similar method of image depth analysis was used; however, two small cameras were used for image acquisition. From these devices a stereo image is obtained and then it creates a depth of space, and subsequently generates appropriate sounds.

In the last years, various research works have addressed such challenges in their attempt to gain a higher level of functioning in the environment and to simplify the navigation of the blind people. The state of the art offers a wide range of electronic wearable device and systems, designed to provide a sensorial substitution to the human vision [17,18,19,20,21,22].

## 3. 3D Sensors

There are many types of 3D sensors. Depending on the application, different technologies are used. The technology used is associated with some of the advantages and disadvantages of the sensors. The work focuses mainly on sensors that can return data in the form of depth maps, or 3D sensors, which could be used in the construction of a device “visualizing” obtained data using sounds, vibrations or other stimuli to which blinds’ perception are capable. Selected types of desirable sensors are ‘time of flight’ sensors, structural light sensors and stereovision systems [23].

### 3.1. Time of Flight Sensors

The Time of Flight (ToF) sensors send a light pulse and measure the time from sending this pulse to returning its reflected rays to the points of the photosensitive array. Return times from different points give a depth map. This is a general idea of the operation of this type of sensors. To reduce interference from the environment and to make the measurements more discreet, the light pulse is emitted in the infrared. In practice, three measurement methods are distinguished: the direct measurement method, the phase shift measurement method and the gated measurement method [11,23].

The direct measurement method is closest to the general idea of ToF sensors. A light pulse (having a duration for instance 10 ns), is emitted, and since then the matrix is ready for return light. In digital circuits, waiting time is measured (separately for each pixel of the image). In points where the reflected light reaches the measurement is stopped [11].

Distance calculations at each point of the matrix based on the flight time can be performed using a very easy equation:

(1) $$D=\frac{1}{2}\Delta t\cdot c$$

where: *D*—measured distance [m]; Δ*t*—trip time [s]; *c*—speed of light [m/s] in the measured center.

If two of the same type sensors are used in one area, at the same time, they can interfere with their operation. In practice, assuming that the duration of the pulse is very short, the pulse path is also relatively short, and the operating frequency is relatively low (e.g., 30 frames per second), there is little probability that the sensors will send impulses that will coincide with each other (unless they use a series of pulses per one frame—then the probability increases) [11].

The method of measuring the phase shift consists not so much in measuring the time in which the reflected light reaches the receiver but measuring the phase shift between the modulated signal emitted by the transmitter and the signal received by the detector [11,16]. Phase shift analysis allows you to determine the time of beam travel. The measuring range of such a sensor is related to the length of the modulated wave (the wavelength of the light is constant, close to the infrared, while the amplitude of this wave is modulated) [11].

In the case of the gated measurement method, a shutter is used which opens the sensor to receive a light pulse at a given time, and thus from a given distance [23]. This approach has an interesting feature—it allows you to capture the image from a selected distance, bypassing the interference that could degrade the quality of the image read. As an example, mist can be given here, which would cause an earlier reflection of the light pulse; however, the shutter is only open to more distant objects, so earlier reflections will not introduce such significant disturbances.

### 3.2. Structured Light Sensors

The basic principle of operation of sensors using structural light is the projection of a known light pattern on the scanned space, reading a distorted pattern using a calibrated camera/camera and calculating the depth map based on distortions [11]. In practice, structural light sensors project the pattern using projectors or lasers. The projected structures are usually stripes, although there are also other patterns, for instance, the spot in the case of the Kinect sensor (which although it is not a typical structural light sensor can be classified in this group) [11,23]. Depending on the projection method (also hardware limitations) and needs, different types of light are used. In the case of “home” sets, often consisting of multimedia projectors, visible light is projected. This approach makes it difficult to apply the method when there is additional ambient light. Infrared light (e.g., 850 nm) is often used in sensors dedicated to such applications. Such light is not visible to human eyes and (in certain frequency ranges) it does not occur in such large intensities in the environment as visible light.

In the case of 3D scanners, which are used to scan stationary objects, the collection of information about the entire field of view can be carried out using multiple frames and a slowly moving laser line. Such a method is unacceptable in the case of a sensor that scans the depth map of the dynamic scene, which changes quickly, where the readings should be at least a few frames per second (for the needs of the prototype constructed and discussed in this work). The next step after the projection is to take a picture (frame) distorted by the spatial objects of the pattern. If the sensor uses infrared light, unnecessary components are removed by means of optical filters so that the ambient light does not introduce disturbances to useful information.

The captured frame is subjected to computer image analysis. The analysis deals with the detection of distorted shapes and the calculation of depth maps [11]. Due to the need for image analysis, structural light technology requires more computing power than ToF technology. A part of 3D scanners/sensors analyzes images and generates a depth map using a processor embedded in the device, which relieves the processor of the computer that uses the already prepared depth map, returned by the sensor. This type of mechanism was used in the Kinect sensor [16]. This allows the sensor to be used even by computers that do not have very high computing power.

An intercepted image (frame) is transferred to the computer image analysis. The analysis deals with the discovery of distorted shapes and calculating the depth map [13]. Since it is necessary to analyze images the technology of structural light requires more computing power than the ToF technology. Some 3D sensors analyze images and generate a depth map using the processor embedded in the device, which relieves the computer’s processor which uses the completed depth map, returned by the sensor. Such a mechanism was used in the Kinect sensor [23]. This allows the sensor to be used even by computers with low computational capability.

### 3.3. Comparison of Sensor Capabilities

The following comparison does not focus on specific models of 3D sensors but is designed to compare some of the characteristics of 3D sensors using one of the three technologies.

Computational complexity—the highest level of computing power is required by stereovision systems. Structural light sensors are next. The smallest computing power needs ToF sensors. The stereovision systems require the largest calculation costs due to advanced image analysis. Sometimes, additional processors are used, dedicated to the analysis of stereoscopic images which relieves the processor of the computer using the sensor. Structural light sensors also require some computing power, although usually not as large as stereoscopic systems. In some sensors, calculations are performed by an internal processor. The sensors with the least demand for computing power are ToF sensors that do not require complex image analysis, and the necessary calculations are often performed by the internal sensor processor.

Operation under low light—poor lighting is no obstacle for ToF sensors and structural light sensors. These sensors actively illuminate the stage for results. Poor lighting is an obstacle to stereovision systems that use ambient light. When the ambient light is weak, the quality of the images decreases, and thus the correct analysis of the images becomes more difficult. When there is no ambient light, it is impossible to operate the system, unless it additionally brightens the stage.

Operation in strong ambient light—the situation presented above is reversed in the case of strong, direct sunlight. Such light can make it very difficult or impossible for ToF sensors, but the structural light sensors have the most difficulties. The strong insolation of the stage does not cause much difficulty in acquiring images from stereoscopic systems.

## 4. Elements Used in the Device

Among the used elements, we can distinguish several key ones, the operation of which is based on the entire device discussed in this work. In addition to them, there are also elements that are less significant but needed, such as a bicycle helmet, memory card, cables and plugs, TV holder for the Kinect sensor, Raspberry Pi housing, assembly materials and a resistor necessary for reworking the Kinect sensor.

### 4.1. Xbox Kinect v1

**Figure 1:**
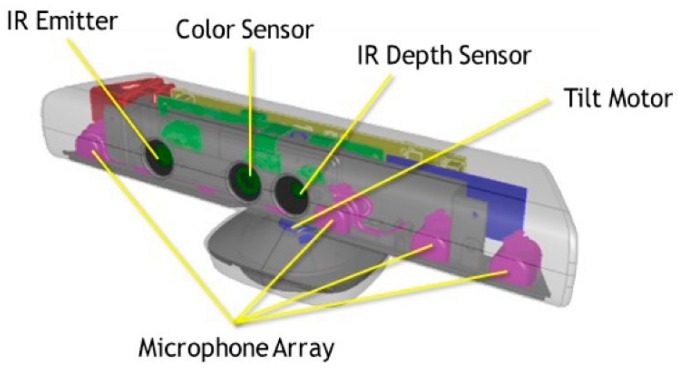
Xbox Kinect with selected sensors. IR Emitter, Color Sensor, IR Depth Sensor (element of structural light sensor), Tilt Motor, Microphone Array. The image does not include the accelerometer [24].

As mentioned before, Xbox Kinect is a 3D (but not only 3D) sensor classified as a structural light sensor because the basis of its operation is the projection of a known pattern (pattern of spots). Kinect is a Microsoft product, but it is based on PrimeSense sensor technology. It was created as a game controller. Due to mass production, it has become cheap and therefore available to many people. It quickly began to be used as a 3D scanner or a 3D sensor for robots. Details of the sensor’s operation are not publicly available, which is why most of the information about the sensor’s exact operation is speculation based mainly on patent documents. The device is equipped not only with a 3D sensor (i.e., a laser projector, infrared camera and optical system—Figure 1), it also has a standard RGB camera, accelerometer and microphone array. In addition, the base is equipped with a motor that drives the sensor inclining system [23].

The viewing angles of the sensor (depth camera and RGB camera) are 43° vertically and 57° horizontally. The vertical viewing angle was additionally “widened” by means of a mechanical sensor inclining system of 27° downwards and upwards. The system is not adapted to continuous operation, but only to periodically adjust the angle of inclination [23].

There is a special overlay—Nyko Zoom—that modifies the optics of the Kinect sensor, by means of which a wider horizontal (and probably vertical) viewing angle can be achieved.

**Figure 2:**
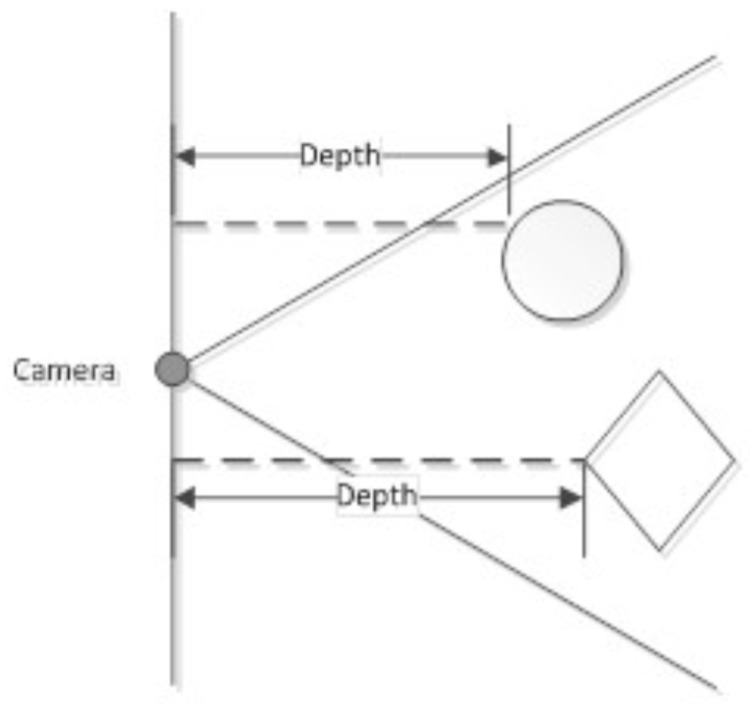
The way the distance is represented by the sensor. The distance is measured from the plane perpendicular to the optical axis of the infrared camera lens [23].

The depth map returned by the sensor has dimensions of 640 by 480 pixels by default. The infrared sensor has a physical resolution of 1280 by 1024 pixels. The depth value (using the freenect library) is returned as an 11-bit number (it is possible to transmit 16-bit depth, but may cause bandwidth problems), where 2047 are distances beyond the sensor range or depth values that could not be read. The depth values of individual points of the field of vision are returned in distances measured not from the lens but from the plane perpendicular to the optical axis of the infrared camera lens (Figure 2).

Direct sunlight prevents the Kinect sensor from reading distances. Fragments of surfaces that are not directly exposed to sunlight can usually be correctly read (or read with defects). The Kinect sensor is therefore not the best device to be used outdoors, but for other reasons has been chosen as a prototype sensor. First of all, due to the low cost of the sensor, the available libraries allow you to use the sensor in a simple way in the Linux system and due to the relatively low power consumption (2.25 W). The device is powered by voltages 5 V (from the USB interface) and 12 V from an additional power supply.

### 4.2. Other Elements

#### 4.2.1. Raspberry Pi 1 Model B+

Rasoberry Pi is not so much a microprocessor as a miniature, cheap computer. It is equipped with general purpose input/output connectors (thanks to which it can easily communicate with other electronic circuits) and many other standard ports, for instance USB, HDMI, Ethernet and minijack (which served as the output of the sound card to which the headphones were connected) prototype of the device discussed in the work).

The SoC BCM2835 multimedia processor manages the entire device. The processor’s SoC type determines the construction of the rest of the device—most of the systems, such as operating memory, GPU, CPU, northbridge and others are built into one microchip. The processor is based on the ARMv6 architecture. The clock frequency of the CPU is 700 MHz. The system can use 256 MB (512 MB for the 1 B+ model) of memory, some of which is allocated to the graphics card. The proportions in which memory is allocated to particular systems can be changed [25].

The Raspberry Pi 1 Model B+ model used to make the prototype of a blind aid device has 512 MB of operating memory, 4 USB ports, a Micro SD card slot (instead of regular SD cards), a better sound system (less noise), more general purpose pins (40 pins). In addition, the consumption of electricity has been reduced. The Raspberry Pi Foundation recommended system is Raspbian—a Linux distribution based on Debian. In addition, there are several other operating systems available for Raspberry Pi. The prototype of the blind aid device described in this paper uses the Raspbian operating system. The device is powered by 5 V with a micro USB connector. The dimensions of Raspberry Pi are 85.60 mm at 56.00 mm and 21.00 mm. The dimensions of the device in the housing are not much larger.

#### 4.2.2. Power Supply—Power Bank

The maximum capacity of the Li-ion battery declared by the manufacturer is 8400 mAh at a 5 V operating voltage. The power bank has two USB charging sockets that can supply 1 A and 2.1 A current to the charging devices (in case of this work to the prototype of the booster the blind). The Raspberry Pi computer is connected to the socket with a maximum current of 1 A, while the power of the Kinect sensor is connected to the 2.1 A socket. The energy bank is charged via the micro USB socket and the maximum charging current is 1 A. The fully charged battery allows the device to operate for 3 h.

#### 4.2.3. Headphones

These are earphones but not in-ear headphones, thanks to which they do not cut off the blind person from the sounds of the surroundings. In the set, three rubber pads are added for a better strapping of the ear mucus. The most important headphone data declared by the manufacturer are: frequency response: 18–22 kHz, sensitivity 107 dB, speaker diameter 15 mm, impedance 32 Ω, and max input power 30 mW.

The total weight of all elements included in the prototype is 480 g.

## 5. Design of a Device Supporting the Blind

The idea for the device and its operation was not modeled on any similar device. The device is not an electronic substitute for a white rod, but something like its complement and extension, because it is an imaging device, so it should be used as an addition to a white stick. Whereas neuroplasticity of the cerebral cortex there is a chance to develop the habit of using this device as a substitute for the eye, that will be reflective and will not require much attention. The device should help to “see” the obstacles (more their presence at larger distances than shape), passages or room shapes. It ought to also help to detection of the presence and location of other people without touch, so as to avoid being hit by a white cane.

All elements of the device were mounted on a bicycle helmet. In this way you can avoid uncomfortably running wires that are easy to hook and pull.

### 5.1. The Principle of Device Operation

As point out before, the device task is to provide a depth map read by the sensor. The map is transmitted to a user using stereo headphones and several sounds (tones with exact frequencies). Sound management is done with software installed and software written right on the Raspberry Pi. The computer links with the sensor via the USB port, and with the headphones using a built-in sound card and a 3.5 mm jack socket.

#### 5.1.1. Depth Values Returned by the Kinect Sensor

The depth value is given by the sensor as 11 bit non-negative number, which gives us values in the range 0–2047, but it is not quite so. In fact, the zero value does not occur. The sensor at the perpendicular position of the optical axis relative to the horizontal surface of the obstacle, “sees” it only at a shortest distance of 57 cm and returns the value 488. If the object surface is set at an angle it is likely to slightly reduce the distance. The supreme value (2047) occurs when the object was detected, but no reading correct distance. This means that the value of 2047 always occurs when the distance rate could not be retrieved. This take place when the object was too close, too far away, when the lighting conditions were unsatisfactory at some point, or when the light has been noised by ambient light.

**Figure 3:**
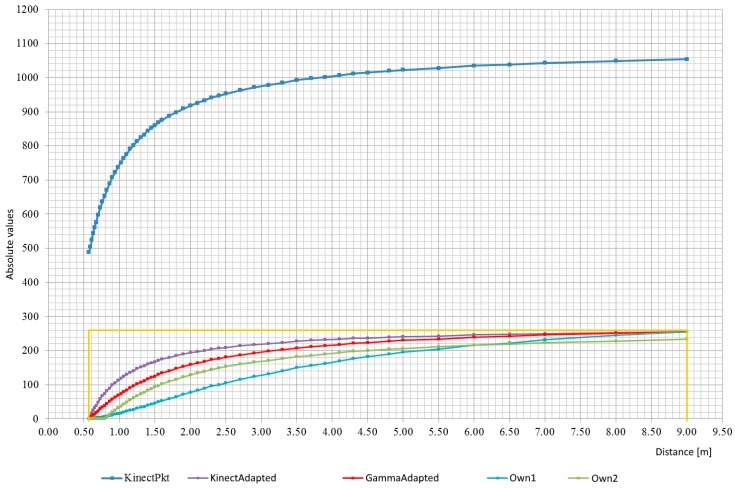
Graphs of functions converting raw distance data (up to 9 m) into useful values in the range of 0–255.

As part of the prototype preparation, measurements of the values returned by the device were made and comparison to the real distance. The program based on the presentation from the OpenKinect website was written for the measurements. The program shows on the screen the distance value read by the sensor in the center of the area of view. For easier pointing, a view of the whole depth map captured was left. Using a measuring cup, the distance between the sensor and the obstacle was measured, which after reading and writing the value was moved away by a reasonable distance. Though the measurements were not very precise, but rather demonstrative, they were precise enough to create characteristics of the sensor. It later helped performing transforming functions that adjust the loudness of the sound to the returned distance (more detailed information on these functions is given below; the way of transmitting the depth map using sounds is given below). You can see that the characteristic is non-linear—Figure 3 blue line.

You can clearly see the high precision and large changes in raw data gather by the sensor with a small variations in distance if the object is nearby. At larger distances, changes in raw values are much minor. However, it is possible to practice such a wide measuring range for the reason that the sampling is stable. Weak precision at more distances is not an obstacle in the use of the sensor on which this article focuses.

Because the Mix_SetPosition (…) function of the SDL_mixer library as the volume/distance value takes the 8-bit parameter (so the max value is 255—silence). It was required, therefore, to process raw values into a functioning range and a slight more linear characteristic using appropriate functions.

Figure 3 presents charts of functions converting raw distance values, 11-bit to 8-bit values. Different version of the transformation functions turn out possibility to adapt the device to a wide open space or a tight like rooms. Rather, changes in the transforming function (and use the most universal) should be avoided, due to the chance that the brain of the person using the device will get accustomed to one function and will be able to recognize correct distance (to make it easier to add a fixed reference sound) volume, described later in this chapter). The mechanism described is not certain but plausible. You can also try to make the selection of the transformation function dependent on some variable component, for instance, on whether the person is in the building or outside.

The chart labeled *KinectPkt* shows the raw values returned by the sensor. Orange lines limit the field of the graph in which the useful range of the actual distance is located and the distance between the range 0–255 is returned by the sensor.

The graph marked as *KinectAdapted* is a graph showing the values of the function relative to the actual distance values:

(2) $$d_{2}\left(KinectPkt\right)=\frac{KinectPkt-488}{2.215}$$

The function scales only raw values so that they are within the desired range. It is suitable for very tight spaces (for which the Kinect sensor is not suitable).

The graph marked as *GammaAdapted* is a graph presenting the values of the function relative to the actual distance values:

(3) $$d_{3}\left(KinectPkt\right)=\frac{{\left(\frac{KinectPkt}{2048}\right)}^{3}\cdot 36\cdot 256-124}{4.44}$$

This function is based on the function—Gamma, which is placed in the demonstrative source code of the *libfreenect* library. The function has been converted in such way that the returned values are within the preferred range.

A graph marked *Own1* shows the values of the function relative to the actual distance values:

(4) $$d_{4}\left(KinectPkt\right)=\frac{2^{\frac{KinectPkt}{80}}}{36}-1$$

It is an empirical function. It is more appropriate for use in the open air than Function (2) or Function (3).

The chart labeled *Own2* shows the values of the function relative to the actual distance values:

(5) $$d_{5}\left(KinectPkt\right)=\frac{{\left(\frac{KinectPkt}{2048}\right)}^{3}\cdot 36\cdot 256-300}{4.1}$$

It is an empirical function. For the largest real distance to be read, it does not give you total silence. It also “skips” a part of the smallest distances detected by the sensor, in which the volume is maximum. Negative values must be set to zero (possible with conditional instructions).

The functions described above ought to be limited in the program from the top and the bottom by means of conditional instructions so that they do not exceed the range of 0–255. These functions are only proposals that do not close the pool of available functions, but only open it. The usability of the device depends on the transformation function. The key is to create a function that works well under different conditions and ensures the best possible hearing of even small differences in the distance in the entire available range.

#### 5.1.2. Transmission of the Depth Map with Sounds

The depth map is transmitted to the user using sounds—fixed-frequency tones. Higher volume means shorter distance to the obstacle. The loudness distribution depending on the distance provides the above-described transforming functions.

The entire depth map given back by the sensor (640 × 480 px) is split into smaller blocks, which in this work will be called small fields. The operation have to be made due to the fact that it would not be possible or very difficult to transmit such a large amount of information. An additional argument for the division of the image are quite often occurring areas in which the sensor could not be able read the distance and such situation would introduce a confusion in the signal and could build an incorrect map of depth in the user’s mind.

**Figure 4:**
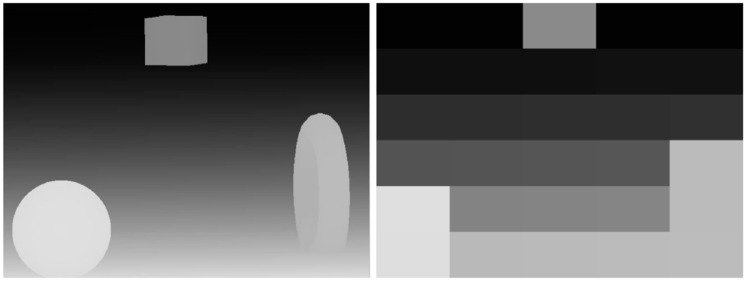
On the **left**, generated depth map for the sample scene. On the **right**, a map of the depth of the scene with a resolution reduced to 6 × 5 px. Images are intended to show only the difference in the reception of different resolutions and do not include the dynamics of the device.

The number of small fields fixes the final resolution of the transmitted depth map. In the prototype made, the depth map forwarded is 5 columns in 6 rows (5 × 6 small fields). This gives 30 small fields (Figure 4). In comparison with the resolution of 640 × 480 px, which gives 307,200 px is not much. There is a good chance to increase the resolution. High resolution has been used in The vOICe program, which by default transmits a map of brightness or depth in 320 × 64 dots resolution.

In the prototype made, the coordinates of the depth point conveyed point depend on the lateralization of the sound signal and the pitch. The depth map is transmitted in the form of a “scan” of five columns from left to right and from right to left (the information in these two “scans” is not the same—this is explained further). Each transmitted column lasts a certain length of time, 200 ms, allowing quite a good listening to the tones. There are 6 small fields in one column (6 rows of depth maps), but not all are transmitted in parallel.

**Figure 5:**
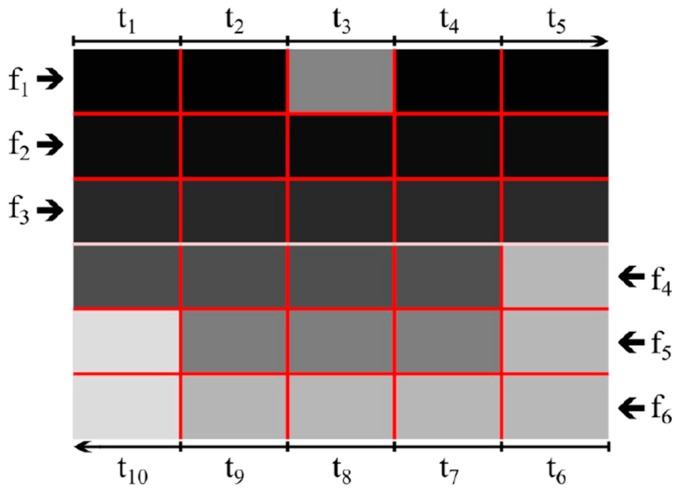
Schematically presented process of sound transmission of the depth. The illustration does not include pauses and reference sound. The arrows marked as fx indicate the “scan” direction and tone, where f1 is the highest frequency tone, and f6 is the lowest frequency tone. Subsequent times of transmitted columns are marked as t_1_–t_10_.

The pitch of individual tones determines at what height there are small fields represented by them—the higher tone is a small field located higher, the lower tone is a small field below. The tones are played in parallel (3 tones, not 6), and the volume of each matches to the distance transmitted by the small field. The scan from left to right is responsible for passing the three upper lines (three higher notes). The scan from right to left is responsible for the transmission of the three bottom lines (three lower tones). A graphic explanation of the above descriptions is given in Figure 5.

Each deep depth map cycle starts with a short tone, always at the same volume, duration 300 ms and frequency 100 Hz, not included in the map. Its purpose is to provide reference volume when a person is once in a quieter, once louder environment.

The sound signal of the transmitted depth map is also appended with pauses for easier identification of particular phases of the message. In the transmission cycle, one can distinguish: phase 1 reference sound with a duration of 300 ms; phase 2 pause (silence) with a duration of 200 ms; phase 3 transmission of 5 columns from three upper rows with a duration of 200 ms (one column) times 5, or 1000 ms; phase 4 pause (silence) with a duration of 200 ms; phase 5 transmission of 5 columns from the three bottom rows with a duration of 200 ms (one column) times 5, or 1000 ms; phase 6 pause (silence) with a duration of 200 ms.

From the described values it is easy to calculate a total cycle time of 2900 ms. This results in a low transmission frequency of around 0.345 Hz. However, it should be noted that before the tone from the next column is reproduced, the depth map values are updated. It can be said that there is a partial refresh rate of 5 Hz (excluding gaps between individual “scans”).

The above times are determined in such a way that the beginner can hear all the sounds and learn what they mean. The next steps in learning how to use the device can be a gradual shortening of the length of individual phases until the minimum times are reached. It is likely that hardware limitations—Raspberry Pi 1 Model B+—would not allow for very short times. This problem is solved with the newer versions of the Raspberry Pi computer, with more computing power. The number of rows (sounds) can be increased. A greater increase in the number of columns results in possibly large, favorable values.

The values of fields are determined as follows: 1—The value of each of the pixels in a small field is computed using a transition table, which stores converted (using one of the transformation functions) at the start of the program distance values. This method was used in the *freenect* library demo code. 2—The values of all correctly read pixels are added together and their number is calculated. Pixels with too large values are discarded. These data serve to average the value later. In addition, the smallest indicated distance value is found among the pixels. 3—If the minimum specified distance value among all pixels in the small area is larger than the defined limit (currently 1.2 m) and if there is at least one pixel with a correctly read value, then the small field is described by means of correctly read pixels. 4—If the above condition was not met (i.e., if a nearby obstacle was detected), the small field is assigned the value of the minimum read distance occurring in the small field area.

You can also use the alternative method of the above algorithm, but it is probably less beneficial to use it. In this method, part of point 2 and point 3 are omitted—the values are not means, and the small field is always assigned the minimum value appearing in its area.

#### 5.1.3. Learning Mode

Since it is not natural to make the view of the height of the view from the height of the sound, it is necessary to give the user the chance to learn this skill. For this purpose, a special program (operating directly on the device) has been made, which should help to learn reflexively depending on the height of the view from the height of the sound. In addition to the program, the person can also learn to recognize the sounds, but this is most likely a much harder learning method.

In order to enable the learning mode, after starting the normal mode, disconnect and immediately connect the Kinect sensor power plug (plug marked with a strip of insulating tape). If the plug-in is connected later than after about seven seconds, the program will not start. The device should then be restarted.

The learning mode works in an analogous way to an ordinary obstacle indicator. Device detects an obstacle situated exactly in the middle area of the sensor’s field of view and signals the smallest distance read in this area from the obstacle using a single tone, which increases in intensity as you approach an obstacle (use the same conversion function, which in normal mode). The height of the tone signaling the obstacle is selected based on the inclination of the sensor. The inclination of the sensor is read using its built-in accelerometer.

If the person bends his head forward (down), then the distance from the obstacle is signaled lower and lower (depending on the angle of inclination). If it tilts the head up, the distance is signaled with ever higher tones. No sound is reproduced if the tilt angle exceeds the field of view angle of the sensor in normal mode, which is 43°, 21.5° down or 21.5° up. It is easy to calculate that for one row (and therefore the tone) it is about 7.17°. Accelerometer readings returned by the *libfreenect* library are returned with a resolution of 0.5°.

The distance refresh rate here is much higher than in normal mode and is 20 Hz (refreshing every 50 ms). The reference sound is played every 80 readings, so every 4 s. It lasts the same as in normal mode—300 ms, and pauses surrounding it after 200 ms.

#### 5.1.4. Selection and Generation of Sounds

In order for the depth map message not to be unpleasant, tones based on the C7 (divided into many octaves) tones shifted by 50 cents up (1/4 tone) were used.

In the selection of sounds, the limitations of human hearing were guided, and so they tried to keep the greatest distances between tones in one “scan”. In addition, two identical sounds could not be heard in a single “scan”.

The basic frequencies of the tones belonging to the C7 token were obtained from the website [26]. Then, the tone frequencies were multiplied by a further power of two to get the frequency of the tones in the higher octaves, and the low frequencies of the tones were divided by successive powers of two to get the frequency of the tones in the lower octaves. The tone frequencies thus obtained have been raised by 50 cents. Of the obtained sounds, 6 frequencies were selected. Selected sounds are presented in Table 1.

The reference tone is in no way associated with the above sounds and has a frequency of 100 Hz, which can be freely changed.

The tones were generated using the Audacity program. The amplitude of the generated sounds has been set to 0.2. The sounds have been matched to each other using the boost slider (slider on the left side of each track). The gain for f = 67.32 Hz was set to +8 dB. For f = 169.63 Hz + 5 dB. For f = 403.49 Hz − 4 dB. For f = 479.86 Hz − 4 dB. For f = 806.97 − 2 dB. For f = 2154.33 − 9 dB.

The tones have been exported to WAV files with a sampling frequency of 44100 Hz, although for the device it can be 22050 Hz, which should not cause any problems.

## 6. Functional Tests of the Device

**Figure 6:**
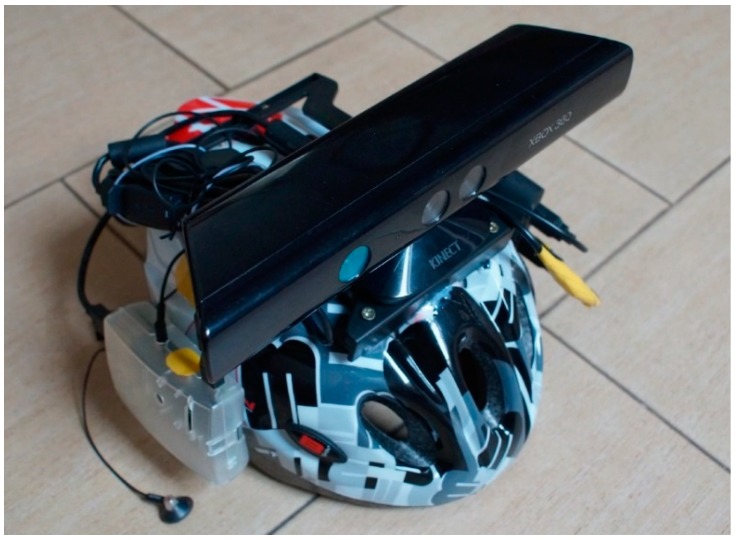
A prototype of our device containing a Raspberry Pi computer with memory card and housing, Xbox Kinect sensor, power bank, sensor holder, headphones and bicycle helmet.

Functional tests of the device (Figure 6) were carried out primarily in the group of people without visual impairment. Such a choice was caused by the lack of access to a larger group of blind people and safety requirements during tests. We chose equation 5 for the main research on the functionality of our device.

**Figure 7:**
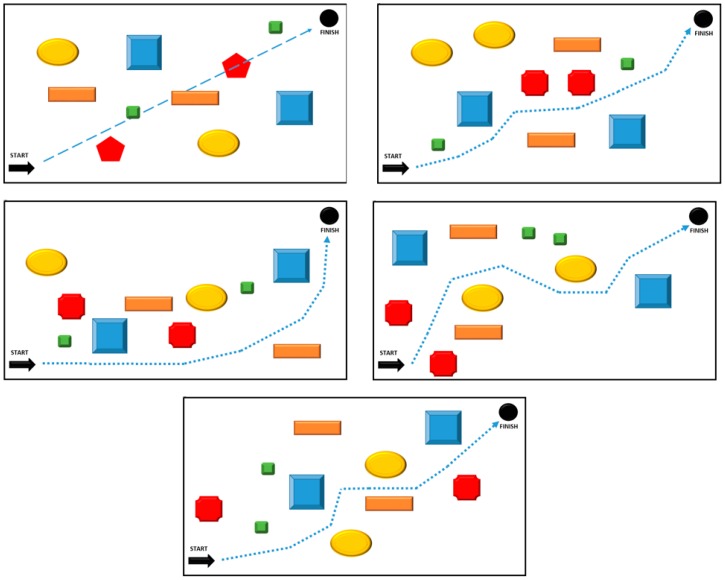
Testing tracks, size of room 20 m × 10 m.

The people who took part in the test were to pass a room with a length of 20 m, using only our blind aid kit. It required tight covering of their eyes with special glasses. The distance to the passage contained 10 obstacles, which had to be avoided, both horizontally and vertically (Figure 7). The passage through the track took place in the “without learning” and “with learning” mode. 176 people took part in the research, each of whom had to go through five different routes, where the difference between the routes resulted from another location of obstacles.

Each of the tested persons was only informed about the location of the starting and ending point before the tests were started. After covering the eyes, the obstacles were set according to the previously established scheme. Of course, the tested people did not have knowledge about the adopted patterns of obstacles for individual routes. The task was to pass the route from the starting point to the end point without colliding with any obstacle. Each collision reduced the parameter, indicating the effectiveness of the correct transition by 1 percent. A collision could have occurred with a given obstacle several times. The transition time was counted from the moment of leaving the starting position, until reaching the final position. Collisions with obstacles did not stop time. Table 2 presents the results of the transition of all 176 people through five different routes in “no learning” mode. Transition efficiency calculated for 176 people on the basis of all correctly avoided obstacles, among all obstacles on the track.

Table 3 shows the results of the effectiveness of users’ transition through selected routes, but this time in “learning” mode. This mode consisted in the possibility of using the built-in learning mode in the device, which allowed for better assimilation of generated sounds and better discrimination between spaces scanning. Each of the tested people had the opportunity to learn the device only once. Such a requirement assumed generating reliable results for all users of the system.

By analyzing the results, it is easy to see that the device requires learning. Each user felt more confident after the learning stage. It is true that the passage times of a 20-m section are not very satisfactory, but as users become familiar with our system, the times have dropped significantly. Different transition times and different transition efficiency were also due to the characteristics of the obstacles placed on the route. The biggest problems were caused by obstacles lying on the ground, requiring the user to turn his head. The remaining obstacles (lateral and placed from the top at the level of the head) after the learning stage were omitted almost every time. One should think about how people who are visually impaired will behave. Such tests will certainly allow for real determination of the capabilities of the device supporting the blind.

## 7. Conclusions and Further Works

The system of acoustic guidance in the surroundings for blind people, and basically prototype, is the device which can be moderated and developed. This work is just the beginning to create a much better device. The sensor itself has a feature (it does not work in sunlight), which rather does not provide it with a good future in this device, although it has become a 3D scanner accessible to many people. Thanks to such high affordability (especially low price), it was also used in this work.

The function of learning the height of the signal based on the head inclination is innovative. If the device is further developed, there is a good chance to discover newer and more effective methods of transmitting depth maps.

Perhaps the attention from blind people would turn out to be the most developmental for such projects. It is possible that this project will be tested by blind people, which may result in improvements of the prototype, which we will certainly strive for.

## Figures and Tables

**Table 1:** The numbers in the table show the frequency of the frequencies in Hz. The frequencies on the green background are used in the first—“scan”. The frequencies on the blue background are used in the second—lower “scan”.

Tone Mark	Frequency Values [Hz]					
C+	67.32	134.63	269.27	538.63	1077.16	2154.33
E+	84.81	169.63	339.26	678.62	1357.13	2714.27
G+	100.92	201.85	403.49	806.97	1613.95	3227.89
Ais+	119.97	239.93	479.86	959.62	1919.24	3838.47

**Table 2:** Transition results of tested persons on 5 different routes in “no learning” mode.

Type of Test	Route Number				
	Route 1	Route 2	Route 3	Route 4	Route 5
Effectiveness [%]	51	62	62	63	65
Average time [s]	131	160	159	169	172
Standard deviation (for time)	9	13	12	13	10

**Table 3:** Transition results of the tested persons on 5 different routes in “learning” mode.

Kind of Test	Route Number				
	Route 1	Route 2	Route 3	Route 4	Route 5
Effectiveness [%]	91	94	97	98	99
Average time [s]	81	92	103	94	111
Standard deviation (for time)	7	9	12	8	9

## References

[1] Kubanek M., Depta F., Smorawa D. (2017). System of Acoustic Assistance in Spatial Orientation for the Blind. Hard and Soft Computing for Artificial Intelligence, Multimedia and Security. Proceedings of the International Multi-Conference on Advanced Computer Systems.

[2] Pec M., Bujacz M., Strumillo P. Personalized head related transfer function measurement and verification through sound localization resolution. Proceedings of the 15th European Signal Processing Conference (EUSIPCO 2007).

[3] Pec M., Strumillo P., Pelczynski P., Bujacz M. (2006). The Hearing Images–Support Systems of Blind People in the Perception of the Environment.

[4] Moore B. (2003). An Introduction to the Psychology of Hearing.

[5] Buonamici F., Furferi R., Governi L., Volpe Y. (2015). Making blind people autonomous in the exploration of tactile models: A feasibility study. Lect. Notes Comput. Sci..

[6] Hayhoe S. (2012). Non-visual programming, perceptual culture and multimedia: Case studies of five blind computer programmers. Multiple Sensorial Media Advances and Applications: New Developments in MulSeMedia.

[7] Tolman B., Harris R.B., Gaussiran T., Munton D., Little J., Mach R., Nelsen S., Renfro B., Schlossberg D. The GPS Toolkit Open Source GPS Software. Proceedings of the 17th International Technical Meeting of the Satellite Division of the ION.

[8] Velazquez R., Ekuakille A.L., Mukhopadhyay S.C. (2010). Wearable Assistive Devices for the Blind. Chapter 17. Wearable and Autonomous Biomedical Devices and Systems for Smart Environment.

[9] Taguchi Y., Jian Y.D., Ramalingam S., Feng C. Point-plane SLAM for hand-held 3D sensors. Proceedings of the IEEE International Conference on Robotics and Automation (ICRA).

[10] Paudel D.P., Demonceaux C., Habed A., Vasseur P., Kweon I.S. 2D-3D camera fusion for visual odometry in outdoor environments. Proceedings of the International Conference on Intelligent Robots and Systems (IROS 2014) 2014 IEEE/RSJ.

[11] Stefanczyk M., Kornuta T. (2014). Image acquisition RGB-D: Methods. Meas. Autom. Robot..

[12] Al-Naji A., Gibson K., Lee S.-H., Chahl J. (2017). Real Time Apnoea Monitoring of Children Using the Microsoft Kinect Sensor: A Pilot Study. Sensors.

[13] 1Takizawa H., Yamaguchi S., Aoyagi M., Ezaki N., Mizuno S., Cane K. (2015). Kinect cane: An Assistive System for the Visually Impaired Based on the Concept of Object Recognition Aid. Pers. Ubiquitous Comput..

[14] Vera P., Zenteno D., Salas J. (2014). A smartphone-based virtual white cane. Pattern Anal. Appl..

[15] Dang Q.K., Chee Y., Pham D.D., Suh Y.S. (2016). A virtual blind cane using a line laser-based vision system and an inertial measurement unit. Sensors.

[16] The project of the Virtual Acoustic Space. http://www.iac.es/proyecto/eavi/english/investigacion.html.

[17] Tapu R., Mocanu B., Zaharia T. (2018). Wearable assistive devices for visually impaired: A state of the art survey. Pattern Recognit. Lett..

[18] Malūkas U., Maskeliūnas R., Damasevicius R., Wozniak M. (2018). Real time path finding for assisted living using deep learning. J. Univ. Comput. Sci..

[19] Ramadhan A.J. (2018). Wearable Smart System for Visually Impaired People. Sensors.

[20] Orujov F., Maskeliūnas R., Damaševičius R., Wei W., Li Y. (2018). Smartphone based intelligent indoor positioning using fuzzy logic. Future Gener. Comput. Syst..

[21] Elmannai W., Elleithy K. (2017). Sensor-Based Assistive Devices for Visually-Impaired People: Current Status, Challenges, and Future Directions. Sensors.

[22] Stoll C., Palluel-Germain R., Fristot V., Pellerin D., Alleysson D., Graff C. (2015). Navigating from a Depth Image Converted into Sound. Appl. Bionics Biomech..

[23] Zhang Z. (2012). Microsoft Kinect Sensor and Its Effect. IEEE Multimed..

[24] Kinect for Xbox 360 and Kinect for Windows (KfW) v1 specs. https://zoomicon.wordpress.com/2015/07/28/kinect-for-xbox-360-and-kinect-for-windows-kfw-v1-specs/.

[25] Upton E., Halfacree G. (2013). Raspberry Pi. User’s Guide.

[26] Tone Frequency Table. http://www.fizykon.org/muzyka/muzyka_tabela_czestotliwosci_tonow.htm.

